# Integrated mRNA and microRNA expression analysis of root response to phosphate deficiency in *Medicago sativa*

**DOI:** 10.3389/fpls.2022.989048

**Published:** 2022-09-13

**Authors:** Zhenyi Li, Zongyong Tong, Feng He, Xianglin Li, Juan Sun

**Affiliations:** ^1^Key Laboratory of National Forestry and Grassland Administration on Grassland Resources and Ecology in the Yellow River Delta, College of Grassland Science, Qingdao Agricultural University, Qingdao, China; ^2^Institute of Animal Sciences, Chinese Academy of Agricultural Sciences, Beijing, China

**Keywords:** *Medicago sativa*, phosphate deficiency, transcriptome, miRNA, miRNA-targeted gene

## Abstract

The deficiency of available phosphate significantly limits plant growth and development. This study sought to investigate how alfalfa (*Medicago sativa*), a high-yielding and high-quality forage widely cultivated worldwide, responds to phosphate deficiency stress by integrating transcriptional and post-transcriptional data. In this study, 6,041 differentially expressed genes (DEGs) were identified in alfalfa roots under phosphate deficiency conditions. Furthermore, psRNATarget, RNAhybrid, and TargetFinder were used to predict the target genes of 137 differentially expressed miRNAs (DEMs) in the root. In total, 3,912 DEGs were predicted as target genes. Pearson correlation analysis revealed 423 pairs of miRNA-mRNA regulatory relationships. MiRNA negatively regulates mRNA involved in regulatory pathways of phosphate deficiency responses in alfalfa. miR156e targeted *squamosa promoter-binding-like protein 13A* (*SPL13*), miR160c targeted *auxin response factor 18* (*ARF18*), and miR2587a controlled glycolysis and citrate cycle *via Phosphoenolpyruvate carboxykinase* (*ATP*) (*PCKA*). Novel-miR27 regulated *SPX domain-containing protein* that controls phosphate transport in alfalfa root, novel-miR3-targeted *sulfoquinovosyl transferase SQD2* controlled sulfolipid synthesis and *glutathione S-transferase* (*GST*; mediated by miR169j/k and novel-miR159) regulated glutathione metabolism. miR399l regulated *auxin-responsive protein SAUR72* involved in IAA signal transduction, while *abscisic acid receptor PYL4* (regulated by novel-miR205 and novel-miR83) participated in ABA signal transduction. Combined miRNA-mRNA enrichment analysis showed that most miRNAs regulate the phosphate starvation response of alfalfa by modulating target genes involved in carbohydrate metabolism, sulfolipid metabolism, glutathione metabolism, and hormone signal transduction. Therefore, this study provides new insights into the post-transcriptional regulation mechanism of phosphate deficiency responses and new perspectives on phosphate assimilation pathways in alfalfa and other legumes.

## Introduction

Phosphorus is an essential macronutrient for plants and participates in various physiological processes of plant metabolism, growth, development, and reproduction (Lopez-Arredondo et al., 2014). Orthophosphate is the sole form of phosphate (Pi) absorbed and utilized by plants in the field. However, about 70% of the global arable land including acidic and alkaline calcareous soils, is deficient in available Pi (Kirkby and Johnston, 2008). Otherwise, orthophosphate is rapidly converted to insoluble and organic phosphorus, and depletes available Pi in the soil, exposing plants to Pi deprivation stress (Lopez-Arredondo et al., 2014; Liu, 2021). Conversely, plants have evolved highly controlled mechanisms to maintain Pi homeostasis and promote phosphorus circulation *in vivo* (Rouached et al., 2010). For example, plants enhance Pi availability from the soil through remodeling root architecture, inducing Pi transporters, secreting organic acids and phosphatases, symbiosis with arbuscular mycorrhizal fungi (AMF), storing phosphate, remobilizing phosphate, and optimizing the metabolic process of phosphate utilization (Rouached et al., 2010; Lopez-Arredondo et al., 2014; Lambers, 2022).

Plants acquire more Pi by suppressing the development of the primary root and increasing the length and density of lateral roots and root hairs, resulting in a root system with a larger surface area (Lynch, 2011; Liu, 2021). Similarly, alfalfa (*Medicago sativa*) adapts to Pi deficiency by increasing the number and length of root hairs (Vance et al., 2003; Li et al., 2022). Pi transporters including PHT1, PHT2, PHT3, and PHT4 are abundantly expressed under Pi deprivation conditions, thus promoting the uptake and transport of Pi by roots (Liu et al., 2011). *MtPT6*, a member of the PHT1 family gene from *M. truncatula*, effectively improves Pi acquisition in transgenic *Arabidopsis* (Cao et al., 2020).

Plant roots also secrete organic acids, which enhance Pi absorption (Liu, 2021). The *ALMT* (*Al-activated malate transporter*) and *MATE* (*multi-drug and toxic compound extrusion*) enhance plant tolerance to aluminum toxicity by regulating the exudation of malate and citrate (Liu et al., 2009; Tokizawa et al., 2015). Plants also exude phosphatase into the soil under Pi deficiency conditions to activate organic phosphorus and induce the expression of acid phosphatase (ACP) and ribonuclease genes (Dong et al., 2004; Zhang et al., 2014b). The *M. truncatula purple acid phosphatase* encoding gene *MtPAP1* effectively improves phytate absorption, which was the sole source of phosphorus in *Arabidopsis* cultivated under phosphate-deficient conditions (Xiao et al., 2006). *GmACP1* overexpression increased Pi uptake efficiency in soybean root hairs by about 11%–20% (Zhang et al., 2014a).

MicroRNAs (miRNAs) are a kind of small RNAs, endogenously synthesized non-coding RNAs (~22 nucleotides long) that play critical roles in a wide variety of biological processes (Chen, 2009). miRNA complementarily combines with target genes and dissociates from the single phosphodiester bonds present in the target mRNA molecules (Brodersen et al., 2008; Khraiwesh et al., 2012). Consequently, the target gene with potential silencing is induced by spliced transcription or suppressed translation by loading through RNA-induced silencing complex (RISC) onto ARGONAUTE (AGO; Ha and Kim, 2014). miRNAs play essential roles in maintaining Pi homeostasis in plants (Fujii et al., 2005; Kuo and Chiou, 2011; Lei et al., 2016; Kumar et al., 2017). Some studies have shown that miRNAs are involved in the response of *Arabidopsis* (Hsieh et al., 2009), white lupin (*Lupinus alba*; Zhu et al., 2010), common bean (*Phaseolus vulgaris*; Valdés-López et al., 2010), soybean (*Glycine max*; Zeng et al., 2010; Xu et al., 2013), *M. truncatula* (Jagadeeswaran et al., 2009) and other plants to Pi starvation stress. The response of certain miRNAs (such as miR399, miR827, miR156, miR2111, miR396, miR319, and miR1507) to Pi-deficiency stress in plant has been clarified (Fujii et al., 2005; Hackenberg et al., 2013; Paul et al., 2015; Lei et al., 2016; Kumar et al., 2017). miR399 is specifically expressed under low Pi conditions and can regulate the protein degradation of two Pi transporters (*PHO1* and *PHT1*) by cleaving the *ubiquitin-binding E2 enzyme* encoding *PHO2* mRNA (Aung et al., 2006; Bari et al., 2006). Therefore, miR399 is involved in Pi uptake and root-to-shoot transport in plants (Chiou et al., 2006; Liu et al., 2012).

Alfalfa is a high-quality forage crop widely used globally. In China, alfalfa is mainly distributed in the northwest where most soils are alkaline and calcareous (Guo et al., 2021). Consequently, Pi deficiency and drought limit alfalfa productivity in these soils (Fan et al., 2015). Most studies have separately investigated miRNA and mRNA regulation mechanisms under Pi deprivation at the transcriptional level (Hsieh et al., 2009; Zhu et al., 2010; Wang et al., 2014; Li et al., 2018, 2022; Ding et al., 2021). However, integrating transcriptional and post-transcriptional datasets to uncover the co-regulatory mechanisms is currently an important research focus. A few studies have reported on the combined miRNA and mRNA regulatory mechanisms of responding to Pi starvation in crops, except for alfalfa. This study analyzed the complex response pathways regulated by miRNA and mRNA to obtain a comprehensive mechanism for improving the efficiency of Pi acquisition, recovery, and recycling in roots. Therefore, it provides a reference for assessing the miRNA-mediated regulation mechanism of Pi deficiency response in alfalfa and a foundation for improving the efficiency of Pi absorption and utilization in alfalfa.

## Materials and methods

### Plant material and growth conditions

Alfalfa (*M. sativa* cv. Magnum Salt) was used in this study. Alfalfa seedlings were transferred to a nutrient solution after 8-days of seeds germination and cultured as previously described (Li et al., 2018). Briefly, the seedlings were cultured in normal nutrient solution for 15-days to ensure growth uniformity. The seedlings were then cultured under normal Pi condition (NP, 500 μmol/L KH_2_PO_4_) or Pi deficient condition (LP, 5 μmol/L KH_2_PO_4_) for 20 days. KOH was added to the nutrient solution to adjust the pH to 5.8–6.0. KCl was added in Pi deficient solution to sustain enough K nutrient. Root samples were collected for subsequent mRNA and miRNA transcriptome sequencing analysis. Each treatment had three biological replicates.

### mRNA and miRNA transcriptome data obtained under Pi deprivation conditions

Mid-vegetative alfalfa seedlings subjected to normal or Pi deficiency treatment for 20 days were used for sequencing analysis. Sequencing of small RNA transcriptome data was conducted as previously described (Li et al., 2018). Then, the small RNA transcriptome data were downloaded from the GEO database (NCBI accession number: SRP110842). The mRNA transcriptome data were obtained from the same root sample as the small RNA data. The sequencing and analysis were conducted by Shanghai Majorbio Technology Co Ltd.^1^ Briefly, total RNA was extracted, and its integrity was validated before further analysis. A library was constructed using RNA for each sample. Then, the cDNA libraries were sequenced on the Illumina Hiseq2500/Miseq system (2 × 150 bp), and the obtained sequences were uploaded to the NCBI database (accession number: SRP133551).

### Transcriptomic analysis of alfalfa root under Pi deficiency conditions

Fastp^2^ was used to remove sequencing adapters, low-quality sequenced bases, poly-N sequences, and unknown bases from the raw data. HISAT2^3^ was used to align the clean reads to the reference genome of *M. sativa* cv. Xinjiang Daye (Chen et al., 2020b). RSEM was used to quantify the expression levels of the transcripts (Zhou et al., 2016). The expression quantification results were normalized to transcripts per million (TPM) reads. The DESeq2 was used for differential expression analysis. Differentially expressed genes (DEGs) were identified at a threshold of log|fold change (LPR/NPR)| > 1 and *p* < 0.05. The DEGs were annotated using the related databases including NCBI-NR,^4^ Swiss-Prot,^5^ GO (Gene Ontology),^6^ and KEGG (Kyoto Encyclopedia of Genes and Genomes).^7^ The functional classification of the DEGs, including GO functional enrichment and MapMan^8^ (Usadel et al., 2009) classification, was also conducted.

### Prediction of miRNA target gene

The candidate target genes to be predicted were DEGs obtained from the same samples. Target genes were predicted using psRNATarget^9^ (Dai et al., 2018), RNAhybrid^10^ (Rehmsmeier et al., 2004), and TargetFinder^11^ (Allen et al., 2005). The target genes from the three prediction gene sets were selected as the candidate miRNA-targeted genes of the corresponding miRNA. The distribution of the predicted miRNA-targeted genes was analyzed using TBtools (Chen et al., 2020a).

### Construction of miRNA-mRNA regulatory network

The correlation between miRNA and mRNA expression was analyzed using Pearson correlation to further determine the regulatory relationship between miRNAs and their target genes (Bylesjo et al., 2007). Negatively co-expressed miRNA-target gene pairs were obtained using the value of miRNA-mRNA at a threshold of correlation < −0.8 and *p* < 0.05 (Fan et al., 2021). Cytoscape (version 3.9.1) was used to selectively construct the miRNA-mRNA regulatory network (Shannon et al., 2003). Furthermore, the miRNA-regulated target mRNAs were subjected to GO, KEGG, and MapMan enrichment analyses. The biological processes related to the miRNA-regulated target genes involved in the regulation mechanism of Pi uptake or pathways response to Pi deficiency were also determined. Subsequently, the enriched metabolic pathways or biological processes were visualized.

### Expression validation of miRNA and mRNA using qRT-PCR

In this study, alfalfa seedlings were cultivated with strict adherence the conditions for RNA-seq as described by Li et al. (2018). The root tissue of the seedlings was used for total RNA extraction after they were subjected to 20 days of phosphate deficiency. The total RNA extracted was further used for qRT-PCR assay. Reverse transcription of miRNA was performed by stem-loop method as described in miRNA 1st Strand cDNA Synthesis Kit (by stem-loop; MR101, Vazyme, Nanjing, China). The specific primers for miRNAs reverse transcription and qRT-PCR assay were designed using miRNA Design V1.01 software (Vazyme, China). The U6 spliceosomal RNA of *M. truncatula* was used as internal reference for detection of miRNA expression. The universal reverse primer mQ-Primer-R and miRNA Universal SYBR qPCR Master Mix (MQ101, Vazyme) was used for qRT-PCR detection of miRNAs. The process of reverse transcription of mRNA was performed as described in the cDNA Synthesis Kit (R212, Vazyme). qPCR master mix (Q411, Vazyme) was used for qRT-PCR. *β-actin* was used as internal reference. The specific primers for genes were designed by Primer-BLAST.^12^ The primers used in this assay listed in Supplementary Table 1. In this assay, each sample contained three biological replicates, each comprising three technical replicates.

## Results

### mRNA sequencing data overview

**Figure 1:**
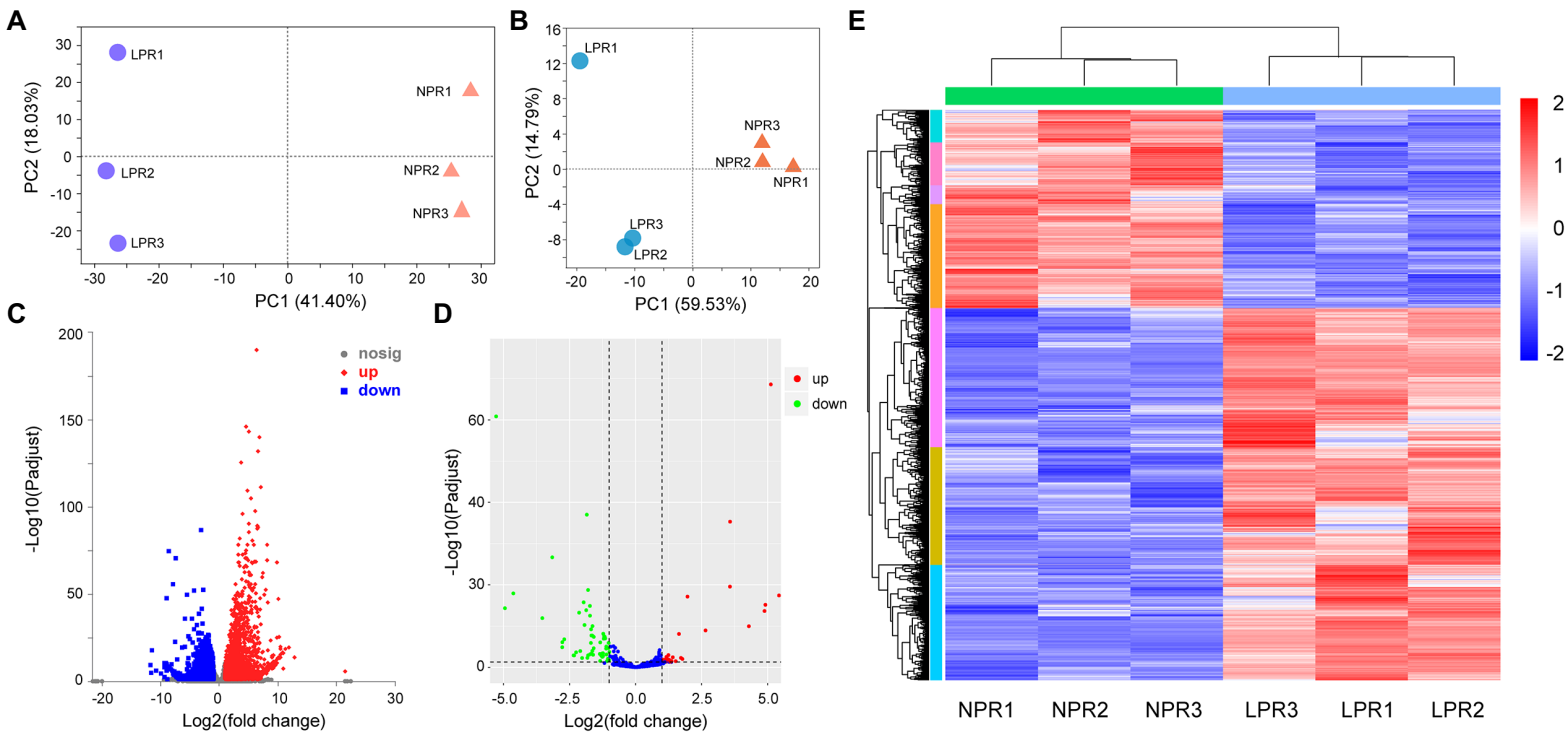
The profiles of differentially expressed genes (DEGs) and differentially expressed miRNAs (DEMs)under Pi deficiency conditions. **(A)** The principal component analysis (PCA) of DEGs under normal and Pi deficient conditions. **(B)** The PCA of DEMs under normal and Pi deficient conditions. **(C)** The volcano of DEGs. **(D)** The volcano of DEMs. **(E)** The profiles of DEGs in different libraries. NPR represents alfalfa seedlings roots under normal phosphate treatment. LPR represents alfalfa seedlings roots under phosphate deficiency treatment. Red or blue box indicates increase or decrease in the fold change value. Full red indicates ≥ 2-fold change. Full blue indicates ≤ −2-fold change.

More than 6G of clean reads were obtained in each library by transcriptome sequencing. After filtering out low-quality reads and sequencing adapters, the Q30 values were greater than 90.92%. About 83.89%–86.38% of the reads were mapped to the alfalfa genome, and 40.29%–42.17% were uniquely mapped (Supplementary Table 2). The principal component analysis (PCA) of the mRNA expression data showed that the first principal component contributed 41.40% of the difference between the two groups (Figure 1A). Moreover, the wide distance between transcriptional data in root under normal Pi treatment (NPR) and under deficient Pi treatment (LPR) indicated low similarity and obvious differences in transcriptome patterns (Figure 1A). The DEGs were determined at a threshold of *p-*adjusted <0.05 and log|fold change (FC)| > 1. A total of 6,041 DEGs contained 3,944 upregulated genes and 2,097 downregulated genes were in the roots (Figures 1C,E; Supplementary Table 3A).

### Functional classification of differentially expressed genes

**Figure 2:**
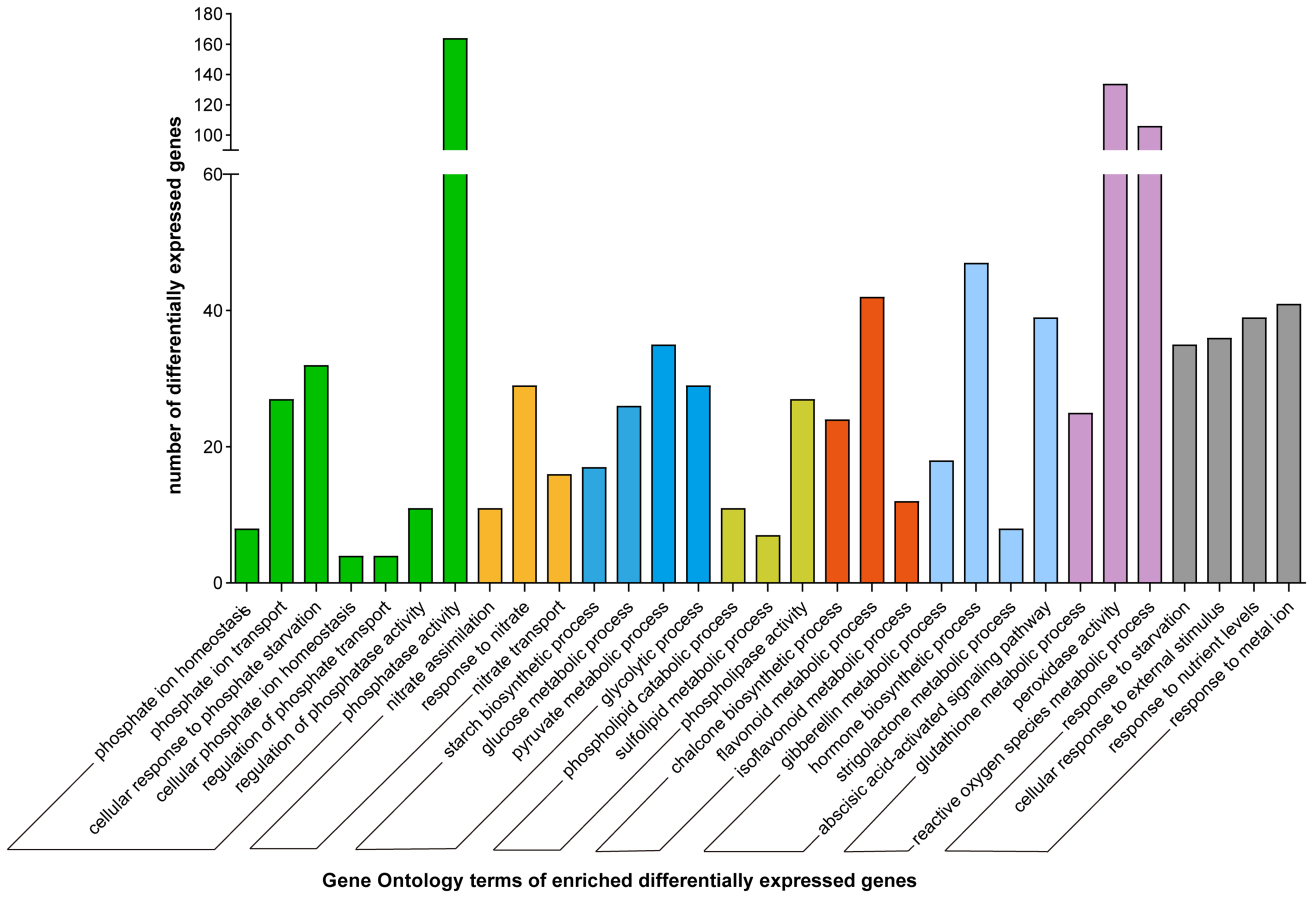
Gene ontology enrichment of differentially expressed genes. Different color represents different functional groups.

The obtained DEGs were further annotated using NCBI-NR, GO, Swiss-Prot, and other databases. GO functional enrichment, and MapMan analyses were used to classify the DEGs. The DEGs were divided into several categories involved in Pi assimilation (phosphate ion homeostasis, GO:0055062; cellular response to phosphate starvation, GO:0016036; phosphate ion transport, GO:0006817), carbohydrate metabolism (starch biosynthetic process, GO:0019252; glycolytic process, GO:0006096), lipid metabolism (phospholipid catabolic process, GO:0009395; sulfolipid biosynthetic process, GO:0046506), cell growth, flavonoid and isoflavonoid metabolism (chalcone metabolic process, GO:0009714; flavonoid biosynthetic process, GO:0009813), and stress resistance (response to extracellular stimulus, GO:0009991; response to oxidative stress, GO:0006979) based on GO annotation (Figure 2; Supplementary Table 3B).

The expression of E2 ubiquitin-conjugating enzyme 24 (UBC24)/PHO2, involved in phosphate ion homeostasis (Bari et al., 2006), decreased by 1.19–1.44-fold in this study (Supplementary Table 3A). The expression of *SPX domain-containing proteins 1* (*SPX1*) to *SPX3*, involved in the cellular response to Pi starvation (Aung et al., 2006), was increased by 2.67–10.08-fold. The expression of *inorganic phosphate transporters 1*–*4* involved in Pi transport involved in Pi transport increased by 2.65–7.08-fold. The expression of *inorganic phosphate transporters 1–3* was increased by 1.37–2.83-fold (Supplementary Table 3A). Moreover, the expression of *inorganic pyrophosphatases 1* (*PPA1*) and *PPA2*, and *purple acid phosphatases 1* (*PAP1*) and *PAP27* increased by >2.77-fold and >1.24-fold, respectively. The expression of *acid phosphatase* (*ACP*) also increased. These results indicate that Pi deficiency significantly affect the activities of phosphorus-related enzymes. Furthermore, the expression of *protein phosphatase 2C* decreased (Supplementary Table 3A), indicating that Pi deficiency stress may inhibit protein phosphorylation. The expression of *phosphate response regulator like 5* (*PHL5*), a *MYB family transcription factor* involved in phosphorus assimilation, was decreased by 1.07–2.89-fold (Supplementary Table 3A).

The expression of phosphoinositol synthase-related gene encoding *type I inositol-1,4,5-trisphosphate 5-phosphatase* was decreased, while the expression of phosphoinositol-forming enzyme *inositol-3-phosphate synthase* was increased (Supplementary Table 3A). Phosphorus is also stored in plants as inositol phosphate, mostly in the form of inositol hexaphosphate. However, some high-valence phosphoinositides contain more phosphorus than inositol phosphate (Hefferon, 2019). Phytic acid is synthesized *via* continuous sequential phosphorylation of the starting substrate glucose-6-phosphate (G-6-P; Raboy, 2001). Glycolysis-related genes were upregulated by 1.49–3.71-fold (*fructose bisphosphate aldolase*), 1.27–1.72-fold (*glyceraldehyde-3-phosphate dehydrogenase*), 1.44–2.95-fold (*pyruvate kinase 1*), 1.76–3.29-fold (*pyruvate decarboxylase*) and more than 3.07-fold (*putative glycerol-3-phosphate transporter 1*; Supplementary Table 3A), indicating enhanced glycolysis.

Furthermore, the expression of genes involved in response to Pi deficiency stress, including *MLP-like protein 28* (1.34–5.89-fold), *pathogenesis-related proteins* (2.86–7.13-fold), and most *peroxidases*, were increased. The expression of several genes involved in glutathione metabolic process, peroxidase activity, and the metabolic process of reactive oxygen species were also induced. The expression of most genes related to the synthesis of chalcone, isoflavonoids, naringenin-chalcone, and anthocyanin was also induced (Supplementary Table 3B).

MapMan enrichment analysis was used to further assess the functions of the DEGs and their related biological processes. The results showed that the DEGs were involved in plant growth regulation, oxidative protection, hormone synthesis and signal transduction, and protein ubiquitination (Supplementary Figure 1; Supplementary Table 3C). Meanwhile, some DEGs were involved in plant growth (cell design, cycle, and development), abiotic stress responses (including heat shock, cold, drought, wounding, and other abiotic stresses), and plant hormone signal transduction (including auxin, ethylene, cytokinin, jasmonate, and salicylic acid). Transcription factor families were also identified, including *ERF*, *WRKY*, *bHLH*, and *NAC* (Supplementary Figure 1; Supplementary Table 3C).

### Target gene prediction analysis

For the miRNA data, the contribution rate of the first principal component to the difference between the two groups was 59.53%, indicating that the two groups of miRNA data were significantly different (Figure 1B). Based on the previous result (Li et al., 2018), the DEMs were screened only at *p* < 0.5 (Figure 1D), and the results showed that 137 DEMs in the roots, including 86 known miRNAs and 51 novel miRNAs were detected (Supplementary Table 4).

**Figure 3:**
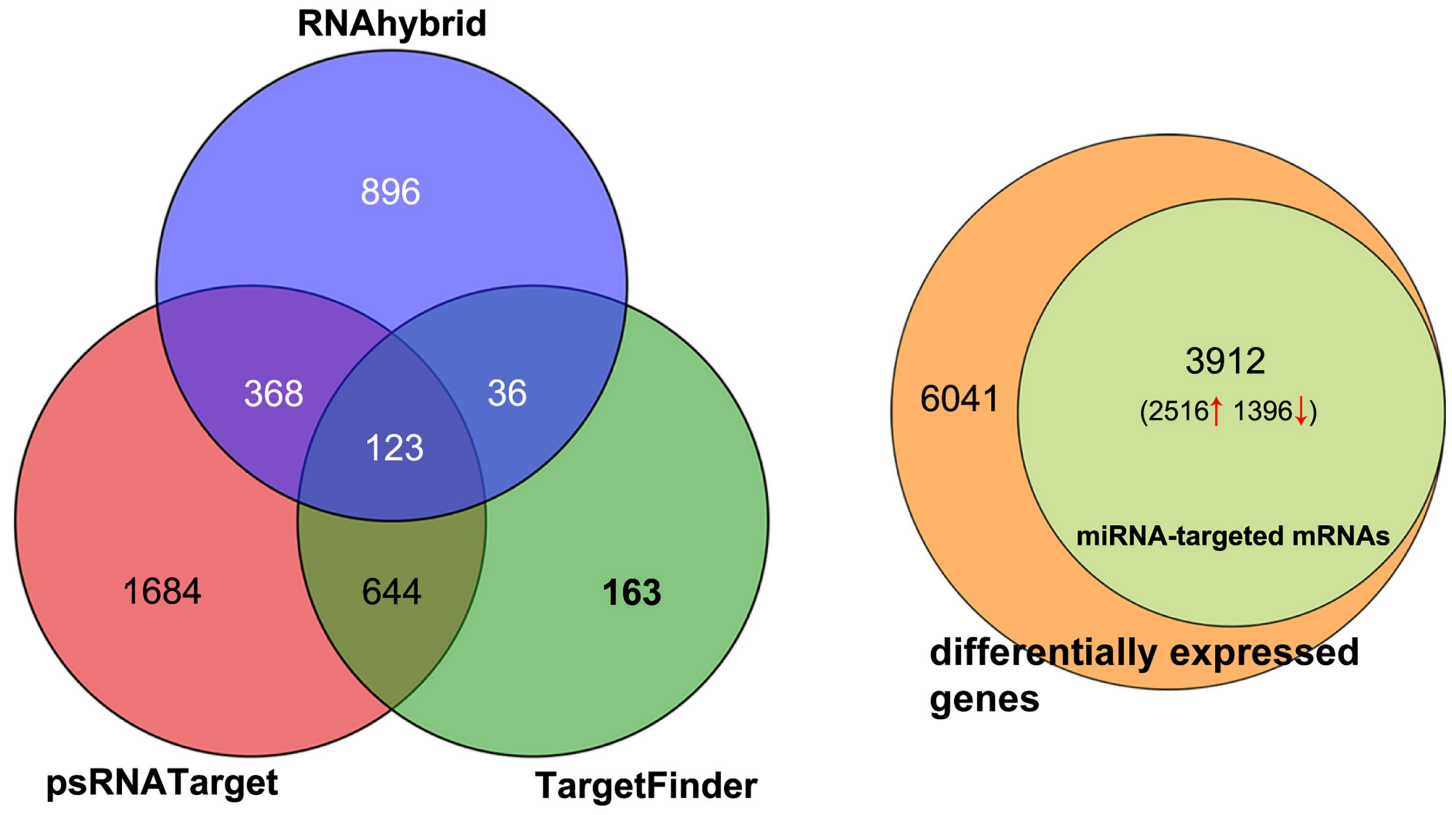
Distribution of miRNA-targeted genes. **(A)** Target genes predicted by RNAhybrid, psRNATarget, and Targetfinder. **(B)** miRNA-targeted mRNAs accounting for total differentially expressed mRNAs.

The target genes of DEMs were predicted using RNAhybrid, psRNATarget, and TargetFinder. The results showed that 1,423, 2,818, and 965 target genes were predicted by RNAhybrid, psRNATarget, and TargetFinder, respectively (Figure 3A; Supplementary Table 5). The intersection of the three sets of results was used to determine the maximum predicted target genes. About 64.76% (3,912) of the DEGs were predicted as target genes, of which 2,516 were upregulated and 1,396 downregulated (Figure 3B).

### Combined expression analysis of miRNA and mRNA

**Figure 4:**
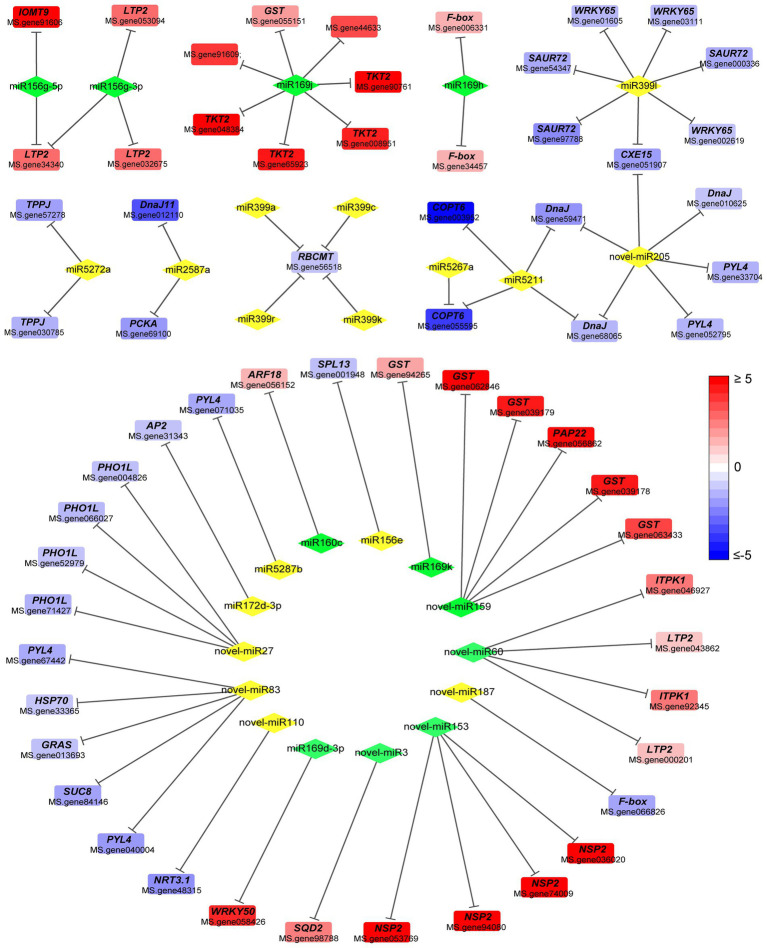
Regulatory network of differentially expressed miRNAs and differentially expressed genes. Diamond and square represent miRNA and mRNA, respectively. Green diamond and yellow diamond represent downregulated and upregulated miRNA, respectively. Line represents negatively regulatory relationship between miRNA and its target. Red or blue square indicates increase or decrease in the fold change value. Full red indicates ≥ 2-fold change. Full blue indicates ≤ −2-fold change. *GST*, *Glutathione S-transferase*; *LTP2*, *non-specific lipid-transfer protein 2*; *TKT2, probable 1-deoxy-D-xylulose-5-phosphate synthase 2*; *F-box, F-box protein*; *IOMT9, isoflavone-7-O-methyltransferase 9*; *SAUR72, auxin-responsive protein SAUR72*; *WRKY65, WRKY transcription factor 65*; *CEX15, carboxylesterase 15*; *DnaJ, chaperone DnaJ-like protein*; *TPPJ, trehalose-phosphate phosphatase J*; *PCKA, phosphoenolpyruvate carboxykinase* (*ATP*); *RBCMT, ribulose-1,5 bisphosphate carboxylase/oxygenase*; *COPT6, copper transporter 6*; *PYL4, abscisic acid receptor PYL4*; *SPL13, squamosa promoter-binding-like protein 13A*; *ARF18, auxin response factor 18 isoform X1; AP2, AP2-like ethylene-responsive transcription factor TOE3*; *PHO1L, phosphate transporter PHO1-like protein*; *HSP70, heat shock 70 kDa protein*; *SUC8, sucrose transport protein SUC8*; *NRT3.1, high-affinity nitrate transporter 3.1*; *GRAS, GRAS family transcription factor*; *NSP2, nodulation-signaling pathway 2 protein-like*; *ITPK1, inositol-tetrakisphosphate 1-kinase 3*; *SQD2, sulfoquinovosyl transferase SQD2.*

Expression correlation analysis between DEMs and target mRNAs in alfalfa roots was performed to identify potential miRNA-mRNA pairs associated with response to Pi deprivation stress. miRNAs negatively regulate the expression of their target mRNAs through mRNA cleavage or translational repression. Herein, miRNA and mRNA had a mutual regulation relationship if: (a) there was a targeting relationship between miRNA and mRNA; (b) the expressions of miRNA and mRNA were negatively correlated with a correlation coefficient < −0.8 and *value of p* <0.05. A total of 423 miRNA-mRNA pairs were identified, of which 187 known miRNA-mRNA pairs and 125 novel miRNA-mRNA pairs were correlated (Supplementary Table 6; Supplementary Figure 2). The combined analysis showed that a miRNA regulated several mRNAs, and several miRNAs also targeted one miRNA. Cytoscape was used to construct miRNA-mRNA regulatory network consisting of negatively correlated miRNA and mRNA pairs involved in Pi starvation response (Figure 4).

Among the known miRNAs, miR156g-5p targeted *isoflavone-7-O-methyltransferase 9* (*IOMT9*), *cytochrome P450 monooxygenase CYP72A65*, *non-specific lipid-transfer protein 2* (*LTP2*) and other genes (Figure 4). Meanwhile, miR156e targeted *squamosa promoter-binding-like protein 13A* (*SPL13*), whereas miR166a targeted *Peroxidase 12-like protein*. miR160c targeted *auxin response factor 18* (*ARF18*), miR2587a targeted *Phosphoenolpyruvate carboxykinase* (*ATP*; PCKA), whereas miR399l targeted *WRKY transcription factor 65* (*WRKY65*) and *auxin-responsive protein SAUR72* (Figure 4).

Among the novel miRNAs, novel-miR110 targeted *high-affinity nitrate transporter 3.1* (*NRT3.1*), novel-miR153 targeted *NPS2 protein-like*, novel-miR159 targeted *glutathione S-transferase* (*GST*) and *purple acid phosphatase 22* (*PAP22*; Figure 4). Meanwhile, novel-miR205 targeted *chaperone DnaJ-like protein* and *abscisic acid receptor PYL4* (*PYL4*), novel-3 targeted *sulfoquinovosyl transferase SQD2* (*SQD2*), novel-miR60 regulated *inositol-tetrakisphosphate 1-kinase 3* (*ITPK1*), while novel-miR83 targeted the *GRAS family transcription factor* (*GRAS*), *PYL4*, and *heat shock 70 kDa protein* (*HSP70*; Figure 4). Some unannotated genes also formed a negative regulatory relationship with miRNAs (Supplementary Table 6).

### The role of miRNA-targeted genes involved in Pi starvation response

Functional enrichment analyses of miRNA-mediated genes, including GO and KEGG enrichment analyses, were performed to integrate the miRNA and mRNA transcriptional datasets of alfalfa roots under Pi deprivation conditions. The miRNAs and their targets involved in the biological processes of responding to Pi deficiency were summarized based on the enrichment of miRNA-targeted DEGs. The GO enrichment analysis showed that Pi is involved in metabolic processes, including cellular response to phosphate starvation (GO:0016036) and phosphate ion transport [GO:0006817 contained *putative SPX domain-containing protein* (MS.gene066027, MS.gene71427, MS.gene004826, and MS.gene52979; Supplementary Table 7A; Supplementary Figure 3)]. Novel-miR27 targeted *SPX domain-containing protein*, also known as *phosphate transporter PHO1-like protein* (*PHO1L*) in *M. truncatula*. In the KEGG pathways, glycolysis (map00010), glycerolipid metabolism (map00561), glutathione metabolism (map00480), plant hormone signal transduction (map04075), vitamin B6 metabolism (map00750), flavonoid and isoflavonoid biosynthesis (map00941 and map00943), and other pathways were enriched (Supplementary Table 7B; Supplementary Figure 4).

**Figure 5:**
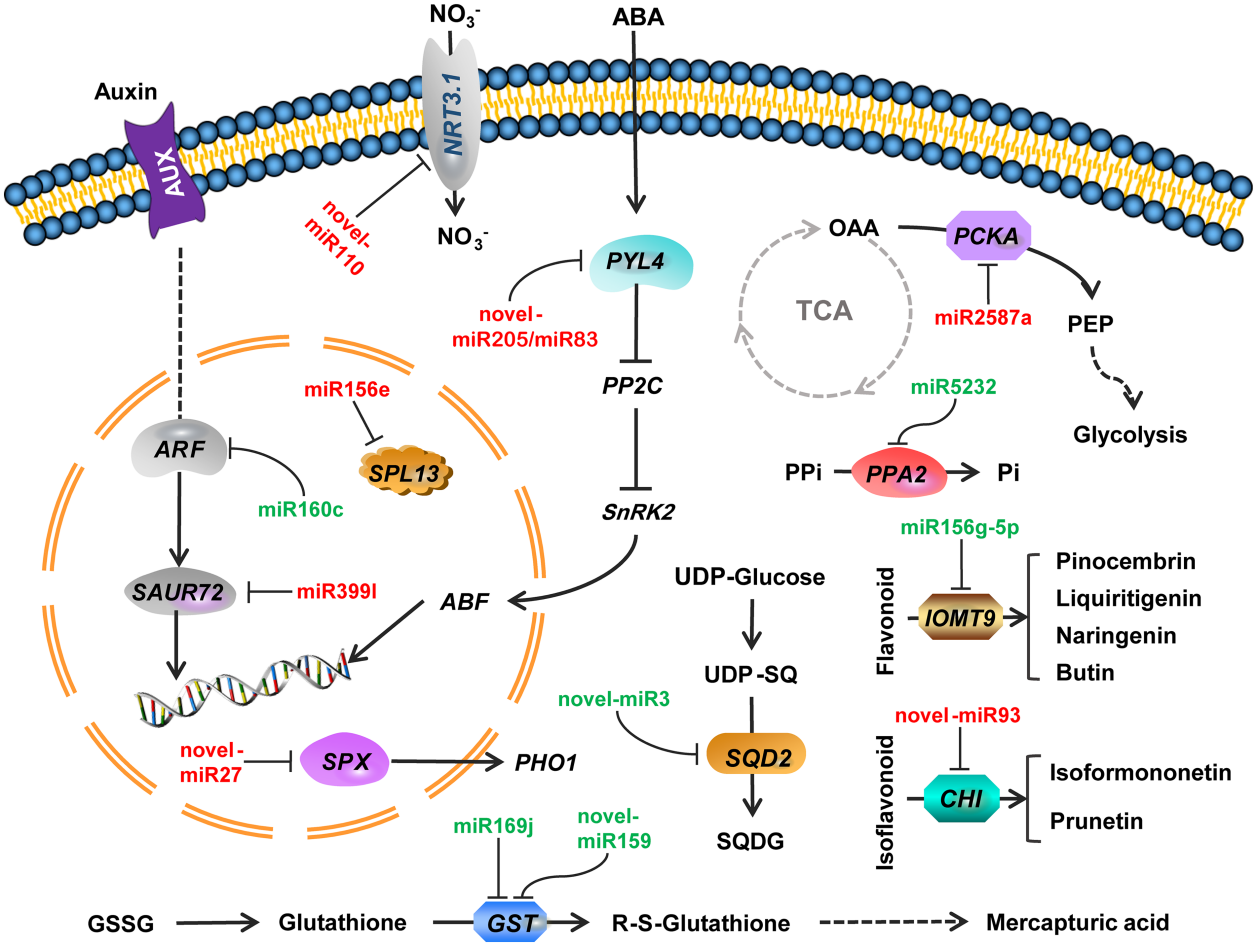
Regulatory pathways of combined miRNA-mRNA responses to Pi deficiency stress in alfalfa roots. Green miRNA represents decreased expression. Red miRNA represents increased expression. Arrows indicate positive regulation. *NRT3.1*, *high-affinity nitrate transporter 3.1*; *ARF*, *auxin response factor 18 isoform X1*; *SAUR72*, *auxin-responsive protein SAUR72*; *PYL4*, *abscisic acid receptor PYL4*; *PP2C*, *Protein phosphatase 2C*; *SnPK2*, *serine/threonine-protein kinase SAPK2*; *ABF*, *abscisic acid-insensitive*; *SPX*, *SPX domain protein*; *PHO1*, *phosphate transporter*; *SPL13*, *squamosa promoter-binding-like protein 13A*; *PPi*, *pyrophosphoric acid*; *PPA2*, *Inorganic pyrophosphatase 2*; *OAA*, *Oxaloacetate*; *PCKA*, *phosphoenolpyruvate carboxykinase* (*ATP*); *PEP*, *Phosphoenolpyruvate*; *SQD2*, *sulfoquinovosyl transferase SQD2*; *SQDG*, *sulfoquinovosyl-diacylglycerol*; *GSSG*, *Glutathione disulfide*; *GST*, *glutathione S-transferase*; *IOMT9*, *isoflavone-7-O-methyltransferase 9*; *CHI*, *Chalcone—flavonone isomerase*.

miRNA-mRNA regulatory pathways were obtained using KEGG functional enrichment analysis. The results showed that miR5232 negatively regulates *PPA* and controls the conversion of pyridoxamine 5-phosphate to pyridoxamine, pyridoxal 5-phosphate to pyridoxal, and pyridoxine 5-phosphate to pyridoxine (Figure 5). More phosphorus is released from the organism through such regulatory mechanisms. Novel-miR3 positively regulates *SQD2*, thus influencing sulfolipid formation, representing how membrane lipids are converted under deficient phosphate conditions (Figure 5). Indole acetic acid (IAA) and abscisic acid (ABA) pathways were enriched in plant signal transduction. The miR399l-downregulated *auxin-responsive protein SAUR72* is involved in IAA signal transduction (Figure 5). Novel-miR159 and novel-miR83 downregulated the *abscisic acid receptor PYL4* and participated in ABA signaling. Novel-miR159 converts glutathione (GSH) into RS-glutathione, which is then converted into glutamate and RS-cysteinyl-glycine. Mtr-miR169j/k negatively regulated *GST* to regulate the glutathione pathway (Figure 5). miR2587a downregulates *PCKA*, and possibly participates in glycolysis and citrate cycle (TCA cycle) by affecting the conversion of oxaloacetate to phosphoenol-pyruvate (Figure 5). Novel-miR93 downregulated *chalcone-flavonone isomerase* (*CHI*), thus affecting the synthesis of pinocembrin, liquiritigenin, butin, and naringenin. miR156g-5p upregulated *IOMT9*, further regulating the synthesis of isoformononetin and prunetin (Figure 5). The expression of miRNA and mRNA is referenced in Table 1.

**Table 1:** The correlation of miRNA and its target mRNA.

miRNA	Log2FC(LPR/NPR) in miRNA	Gene	Log2FC(LPR/NPR) in mRNA	Correlation	miRNA	Log2FC(LPR/NPR) in miRNA	Gene	Log2FC(LPR/NPR) in mRNA	Correlation
miR169j	−6.06	*GST* (MS.gene89257)	1.94	−0.92	Novel-miR159	−1.81	*GST* (MS.gene039178)	4.53	−0.83
miR169j	−6.06	*GST* (MS.gene94265)	1.67	−0.99	Novel-miR159	−1.81	*GST* (MS.gene039179)	4.49	−0.85
miR169j	−6.06	*GST* (MS.gene055151)	1.87	−0.90	Novel-miR159	−1.81	*GST* (MS.gene062846)	5.76	−0.91
miR169k	−2.36	*GST* (MS.gene94265)	1.67	−0.99	Novel-miR159	−1.81	*GST* (MS.gene063433)	3.64	−0.89
miR399l	3.56	*SAUR72* (MS.gene97788)	−2.20	−0.90	Novel-miR205	6.81	*PYL4* (MS.gene33704)	−1.42	−0.87
miR399l	3.56	*SAUR72* (MS.gene000336)	−1.58	−0.92	Novel-miR205	6.81	*PYL4* (MS.gene052795)	−1.37	−0.93
miR399l	3.56	*SAUR72* (MS.gene54347)	−1.58	−0.92	Novel-miR83	2.63	*PYL4* (MS.gene67442)	−1.57	−0.83
miR156g-5p	−2.15	*IOMT9* (MS.gene91606)	5.07	−0.90	Novel-miR83	2.63	*PYL4* (MS.gene040004)	−1.90	−0.86
miR156e	0.45	*SPL13* (MS.gene74056)	−1.15	−0.89	Novel-miR27	1.01	*SPX* (MS.gene066027)	−1.27	−0.98
miR160c	−1.37	*ARF18* (MS.gene056152)	1.38	−0.83	Novel-miR27	1.01	*SPX* (MS.gene71427)	−1.44	−0.85
miR2587a	0.68	*PCKA* (MS.gene69100)	−1.93	−0.98	Novel-miR27	1.01	*SPX* (MS.gene004826)	−1.16	−0.92
miR5232	−1.74	*PPA2* (MS.gene76583)	2.52	−0.86	Novel-miR27	1.01	*SPX* (MS.gene52979)	−1.04	−0.91
Novel-miR110	0.74	*NRT3.1* (MS.gene48315)	−2.11	−0.88	Novel-miR93	0.83	*CHI* (MS.gene029087)	−1.36	−0.99
Novel-miR3	−1.98	*SQD2* (MS.gene001948)	2.44	−0.86					

### qRT-PCR validation of the key miRNA-mRNA pairs

**Figure 6:**
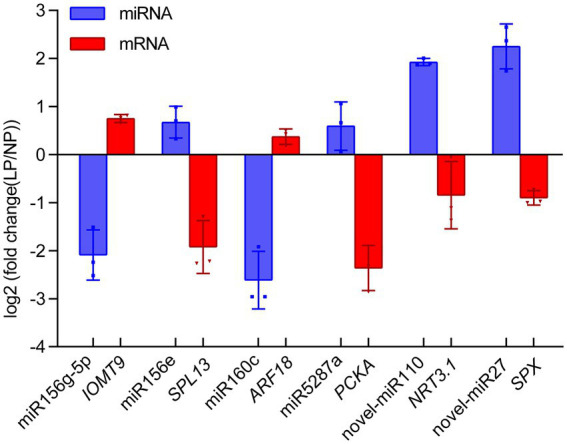
Quantitative real-time PCR validation of miRNA-mRNA interaction pairs in alfalfa roots responding to phosphate deficiency. Blue and red boxes represent the miRNAs and their target mRNA, respectively. Each column represents an average of three replicates, and error bars indicate the standard deviations. *IOMT9*, *isoflavone-7-O-methyltransferase 9*; *SPL13*, *squamosa promoter-binding-like protein 13A*; *ARF18*, *auxin response factor 18*; *PCKA*, *Phosphoenolpyruvate carboxykinase (ATP*); *NRT3.1*, *high-affinity nitrate transporter 3.1*; *SPX*, *SPX domain-containing protein*.

To verify the key miRNA-mRNA pairs involved in phosphate deficiency in roots, we utilized qRT-PCR to analyze the relative expression of miRNA and its target mRNA in alfalfa roots after 20 days of phosphate deficiency treatment. Six miRNA-mRNA pairs were detected. As shown in Figure 6, the expression of miR156g-5p and miR160c decreased by 2.09- and 2.61-fold, whereas the expression of its target gene, *IOMT9* and *ARF18* increased by 0.75- and 0.37-fold. The increased miR156e and miR2587a were negatively correlated with their target genes *SPL13* and *PCKA*, respectively (Figure 6). Notably, the novel miRNAs and their target genes were also detected. Novel-miR110 and novel-miR27 exhibited 1.93- and 2.25-fold increase expressions, whereas their target genes, *NRT3.1* and *SPX*, exhibited 0.84- and 0.90-fold decrease expressions (Figure 6). These results indicated that the expressions of miRNAs were negatively correlated with the expression of their target genes. The results further verified that the expression of miRNA or mRNA shares a more similar trend with miRNA-seq data or mRNA-seq data.

## Discussion

Pi deficiency stress conditions for plants are divided into light, mild and severe (Liu, 2021). Severe and mild Pi deficiency significantly inhibits the growth of plant roots. Mild Pi deficiency (200 μM) increased the total root length, while severe Pi deficiency (20 μM) inhibited root growth of switchgrass (*Panicum virgatum*; Ding et al., 2021). A study also analyzed stress-related mRNAs in alfalfa (*M. sativa* cv. Wudi) after 12 days of Pi deficiency treatment and found some overlapping DEGs (Li et al., 2022). Previous studies have shown that Pi deficiency regulates the expression of miRNA and lncRNA at the transcriptional level in *M. sativa*, *M. truncatula*, and soybean (Wang et al., 2017; Li et al., 2018; Zhang et al., 2021). These findings confirm the presence of phosphate deficiency responding mRNAs and miRNA, thus providing a foundation for identifying key mRNAs related to the regulation of Pi assimilation and Pi starvation response.

In this study, several key genes were detected in alfalfa under Pi deprivation conditions. *UBC24* encodes a binding ubiquitination E2 enzyme, which can affect the expression of low-Pi responding genes and regulate Pi assimilation (Aung et al., 2006; Wang et al., 2020). In this study, *E2 UBC24* was downregulated. *Phosphate response regulator* (*PHR1*), a key *MYB transcription factor*, is also induced under low Pi conditions to maintain phosphorous in plants. SPX protein enhances plant response to Pi deficiency, thus maintaining Pi homeostasis (Wang et al., 2014; Wild et al., 2016; Zhao et al., 2018). *PHR* regulates hypophosphatemia response by modulating the expression of various Pi starvation-responsive genes (Rubio et al., 2001; Wang et al., 2014). *PHR1*, *PHR1-LIKE 1* (*PHL1*), *MYB2*, and several other Pi starvation-responsive genes are upstream regulators of miR399, *PHO2*, *PHTs*, and other Pi-deficiency-responsive genes (Bari et al., 2006; Lopez-Arredondo et al., 2014; Pant et al., 2015). In this study, *PHL5* was decreased by 1.07–2.89-fold, indicating that it potentially participates in Pi deficiency response. *SPX1*–*SPX3* are abundantly expressed in alfalfa under Pi deficiency conditions to regulate the Pi starvation response (Li et al., 2022). *SPX1* and *SPX2* interact with *PHR2* to regulate the *PSI* genes (Wang et al., 2014). *SPX* regulates Pi uptake, and *MtPT* improves Pi acquisition in *M. truncatula* (Cao et al., 2020). Herein, the *aluminum-activated citrate transporter* enhanced the secretion of citric acid and the proportion of available Pi (Furukawa et al., 2007; Liu et al., 2009). Notably, the expression of *aluminum-activated citrate transporter isoform A* was increased in this study. Pi deprivation inhibits the absorption of nitrogen, and NRT3.1 facilitate nitrogen absorption (Okamoto et al., 2006). In the present study, the expression of *NRT3.1* was inhibited, indicating the inhibition of nitrate absorption in alfalfa roots.

GO terms such as carbohydrate metabolism, lipid metabolism, glutathione metabolism, response to stimuli, hormones signal transduction and flavonoid metabolism were enriched under Pi deficiency stress (Rico-Resendiz et al., 2020; Ding et al., 2021; Li et al., 2022). In the present study, phosphate ion homeostasis, flavonoid biosynthetic process, cellular response to external stimulus, glutathione metabolic process, and stress response were enriched under low Pi conditions.

The response of certain miRNAs (such as miR399, miR827, miR156, miR160, miR172, miR2111, miR396, miR319, miR1507) to deficient-Pi stress in plants has been clarified (Fujii et al., 2005; Rouached et al., 2010; Hackenberg et al., 2013; Paul et al., 2015; Lei et al., 2016; Kumar et al., 2017). However, most studies have analyzed miRNA transcription and mRNA transcription separately. Combined analysis can accurately reflect the biological processes related to miRNA-mediated mRNAs. In this study, a complex regulatory network was detected based on conjoint analysis. miR399 is specifically expressed under Pi deprivation conditions, and regulates two groups of Pi transporters (*PHO1* and *PHT1*) by cleaving the *ubiquitin-binding E2 enzyme PHO2* mRNA and enhancing root-to-shoot plant Pi transport and uptake (Chiou et al., 2006; Liu et al., 2012). *PHR1* and *PHL1* may play an upstream regulatory role by binding to the promoter of P1BS, thereby inducing the expression of miR399 (Bari et al., 2006; Zeng et al., 2013). Herein, miR399l targeted the *SAUR* gene, which may represent a mode of action. Novel-miR205 is similar to miR3991, with only two-base differences. Moreover, novel-miR205 has a similar role to miR399, and may be a member of the miR399 family. Novel-miR27, acting on putative SPX domain-containing protein, may be involved in Pi absorption and metabolism.

miR160 acts on the *ARF* gene, and has been detected in alfalfa (Li et al., 2018). Furthermore, miR160 targets *ARF18* in peanuts, and is involved in response to salt stress (Tang et al., 2022). In this study, miR160c-ARF18 was detected in alfalfa and potentially participated in response to Pi deprivation stress. Hofferek et al. (2014) reported that miR171h and *nodulation signaling pathway* (*NSP*) have a negative regulatory relationship based on western blot and qRT-PCR. *NSP2* positively regulates *MtPT4* and participates in Pi absorption in the mycorrhizal roots of *M. truncatula* (Hofferek et al., 2014). Herein, novel-miR153 targeted the *NSP2 protein-like*, suggesting that it may be involved in Pi absorption in alfalfa.

Furthermore, miRNAs that regulate root growth and development also play an important role in *Ammopiptanthus mongolicus* and *Arabidopsis thaliana*, along with miR156, miR160, miR167, miR172, miR319, and other miRNAs (Kuo and Chiou, 2011; Gao et al., 2016). The miR172-AP2 domain protein module is involved in response to N, -P, -K, -NPK stresses in sorghum (Zhu et al., 2021). Overexpression of miR172 in common bean promoted the formation of root hairs and lateral roots by modulating *APETALA2-1* (*AP2-1*; Nova-Franco et al., 2015). In the present study, *AP2* was the target gene of miR172, and its expression was negatively regulated by Pi deficiency stress. miR169 is a potential long-range signal transmitting shoot apical Pi status to roots. miR169 and miR399 have similar roles (Pant et al., 2009). Therefore, miR169 potentially mediates alfalfa response to Pi starvation.

Pi deficiency increases the accumulation of non-phosphorus sugars, such as sucrose and starch (Lin et al., 2014). Sucrose may promote auxin transport and increase root sensitivity to auxin under Pi starvation (Karthikeyan et al., 2007). Secondary system signals (Pi, sucrose, and miR399) are generated in shoots and transported to roots *via* the phloem under Pi starvation. Systematic sensors in the roots sense the shoot-derived system signals (Karthikeyan et al., 2007; Chiou and Lin, 2011). Phospholipids are released from phospholipid reserves through hydrolysis, then converted to sulfolipid or galactolipid during phospholipid deficiency (Pant et al., 2015). Sulfolipid synthesis is controlled by *SQD2* (Yu et al., 2002). The expression of *SQD2* was increased in the present study. Moreover, novel-miR3 regulated *SQD2*, which might control sulfolipid synthesis in alfalfa roots. In the present study, the expression of *monogalactosyldiacylglycerol synthase 2* (*MGD2*) was also increased.

The glutathione metabolic pathway is crucial in plant signal transduction and stress resistance. It also enhances plant response to biotic and abiotic stresses (Cheng et al., 2015). GSH is a thiol-containing bioactive tripeptide with the general structure containing glutamic acid (Glu), cysteine (Cys), and glycine (Gly) linked by peptide bonds (Noctor et al., 2012). GSH scavenges ROS by regulating the activity of the antioxidant enzyme GST (Noctor et al., 2012). Therefore, plants can counteract the excess ROS generated during stress by increasing GSH content in cells. Glutathione is converted to r-s-glutathione *via* glutathione s-transferase (Noctor et al., 2012; Kapoor et al., 2019). The results of this study are consistent with the study results of potatoes (*Solanum tuberosum*) under cadmium stress (Yang et al., 2021). Herein, miR169 negatively regulated *GST* under Pi starvation, inducing a negative effect on glutathione in alfalfa. Glycerol metabolism has a certain regulatory strategy for alfalfa to adapt to Pi deprivation environment.

Signal transduction translates upstream signals into complex downstream reactions. AUX/IAA proteins regulate the transcription of downstream genes by directly inhibiting *auxin-responsive transcription factors* (*ARFs*), thus regulating the IAA signaling pathway (Salehin et al., 2015). In this study, the expression of *ARF18* in the auxin signaling pathway was upregulated. Moreover, miR160c inhibition induced *ARF18* expression. In addition, miR399l and one downstream gene in the auxin pathway inhibited *SAUR* expression. *SAUR* expression was also downregulated, thus affecting auxin signaling. Salt and abscisic acid (ABA) treatments induce miR399f expression, confirming that miR399f participates in plant responses to salt and drought stress (Baek et al., 2016). In this study, novel-miR205 regulated *PYL4*, which is involved in ABA signal transduction. The novel-miR205 was structurally similar to miR399l, differing in only two bases, and thus it may belong to the miR399 family. This study also revealed that miRNA might be involved in response to Pi deficiency through the ABA pathway. Therefore, miR399 may also respond to Pi starvation through other pathways.

Most previous bioinformatics analyses and verifications were based on the *M. truncatula* reference genome. In this study, the genome of *M. sativa* cv. Xinjiang Daye was referenced, greatly complementing previous findings (Li et al., 2018). Therefore, this study provides new insights into understanding the miRNA-mRNA regulatory network of low Pi response. Six key miRNA-mRNA pairs were further verified by qRT-PCR, which confirmed the reliability of the RNA-seq data. Notably, the miRNAs and mRNAs founded in this study may be the key nodes enhancing alfalfa response to Pi deficiency stress. Therefore, the interaction and function of miRNAs and mRNAs should be further verified based on reverse genetics.

## Conclusion

The deficiency of available Pi significantly reduces forage yield and quality in agriculture production. The global analysis of mRNA and miRNA in alfalfa roots under Pi-deficient conditions revealed that candidate genes and related miRNAs are involved in the regulation of Pi deficiency response. miRNA negatively regulates mRNA involved in metabolism pathways. miR2587a targets *PCKA* to regulate glycolysis and TCA cycle metabolisms. Novel-miR3 influences sulfolipid synthesis by regulating *SQD2*. MiR399l-regulated IAA signaling by targeting *SAUR72*. Novel-miR205 and novel-miR83 regulate ABA signaling of *PYL4*. miR169j/k and novel-miR159 mediate *GST* regulation of glutathione metabolism. Novel-miR27 directly affects Pi transport in alfalfa by targeting *SPX*. *PPA* enhances the release of Pi from pyrophosphoric acid (PPi) through miR5232. Furthermore, the key pairs of miR156e targeted *SPL13* and miR160c targeted *ARF18* were found. Combined miRNA-mRNA co-regulation results also showed that alfalfa mainly regulates carbohydrate metabolism, sulfolipid metabolism, hormone signal transduction, glutathione metabolism and Pi assimilation under Pi starvation stress conditions. Therefore, this study provides a basis for exploring the mechanism of miRNA-mediated Pi absorption and assimilation in alfalfa and other legumes.

## Data availability statement

The original contributions presented in the study are publicly available. This data can be found at: NCBI, SRP110842 and SRP133551.

## Author contributions

ZL: experimental design, data analysis, and original draft preparation. ZT: data analysis and validation. FH: resources and data curation. XL: supervision and project administration. JS: project administration and manuscript review. All authors contributed to the article and approved the submitted version.

## Funding

This work was supported by Natural Science Foundation of Shandong Province (ZR2020QC185), China Forage and Grass Research System (CARS-34), Doctoral Scientific Research Startup of Qingdao Agricultural University (6631119038), and The First Class Grassland Science Discipline Program of Shandong Province, China (1619002).

## Conflict of interest

The authors declare that the research was conducted in the absence of any commercial or financial relationships that could be construed as a potential conflict of interest.

## Publisher’s note

All claims expressed in this article are solely those of the authors and do not necessarily represent those of their affiliated organizations, or those of the publisher, the editors and the reviewers. Any product that may be evaluated in this article, or claim that may be made by its manufacturer, is not guaranteed or endorsed by the publisher.

## Footnotes

^1^ http://wwwmajorbiocom/

^2^ https://github.com/OpenGene/fastp

^3^ http://ccb.jhu.edu/software/hisat2/index.shtml

^4^ ftp://ftp.ncbi.nlm.nih.gov/blast/db

^5^ http://web.expasy.org/docs/swiss-prot_guideline.html

^6^ http://www.geneontology.org

^7^ http://www.genome.jp/kegg/

^8^ http://mapman.gabipd.org

^9^ https://www.zhaolab.org/psRNATarget/

^10^ http://bibiserv.techfak.uni-bielefeld.de/rnahybrid

^11^ http://carringtonlab.org/resources/targetfinder

^12^ https://www.ncbi.nlm.nih.gov/tools/primer-blast

## Supplementary material

SUPPLEMENTARY TABLE 1 The primer sequences of miRNA reverse transcription, and miRNAs and target genes for qRT-PCR assay.

SUPPLEMENTARY TABLE 2 Overview of obtained RNA-Seq data from alfalfa root under phosphate deficiency.

SUPPLEMENTARY TABLE 3A Differentially expressed mRNAs in root under phosphate deficiency.

SUPPLEMENTARY TABLE 3B GO enrichment of differentially expressed mRNAs.

SUPPLEMENTARY TABLE 3C The functions of Differentially expressed mRNAs enriched by MapMan.

SUPPLEMENTARY TABLE 4 Differentially expressed miRNAs in root under deficient phosphate.

SUPPLEMENTARY TABLE 5A miRNA targets predicted by RNAhybrid.

SUPPLEMENTARY TABLE 5B miRNA targets predicted by psRNATarget.

SUPPLEMENTARY TABLE 5C miRNA targets predicted by Targetfinder.

SUPPLEMENTARY TABLE 5D All of miRNA targets predicted by RNAhybrid, psRNATarget and Targetfinder.

SUPPLEMENTARY TABLE 6 Correlation analysis of miRNA and mRNA.

SUPPLEMENTARY TABLE 7A The function of miRNA-targeted mRNAs enriched by GO analysis.

SUPPLEMENTARY TABLE 7B The function of miRNA-targeted mRNAs enriched by KEGG analysis.

SUPPLEMENTARY FIGURE 1 MapMan enrichment of differentially expressed genes in alfalfa root under Pi deficiency.

SUPPLEMENTARY FIGURE 2 The expression of miRNAs and their targets mRNA under Pi deficiency.

SUPPLEMENTARY FIGURE 3 Gene ontology enrichment of miRNA-targeted mRNAs under Pi deficiency.

SUPPLEMENTARY FIGURE 4 Kyoto Encyclopedia of Genes and Genomes pathways of miRNA-targeted mRNAs under Pi deficiency.
