# Supplementary material for: Integrated mRNA and microRNA expression analysis of root response to phosphate deficiency in Medicago sativa
Source: Front Plant Sci. 2022 Sep 13;13:989048. doi: 10.3389/fpls.2022.989048 (PMC9513243; doi:10.3389/fpls.2022.989048)
Supplement: SUPPLEMENTARY TABLE 1 — The primer sequences of miRNA reverse transcription, and miRNAs and target genes for qRT-PCR assay. [file Table_1.DOCX]

| Primer name | Sequence (5’-3’) | Gene number | Using for |
| --- | --- | --- | --- |
| miRNA qRT-PCR | | | |
| U6-F | CTTCGGGGACATCTGATAAA | XR_003007418.1 |  |
| U6-R | ATTTCTCGATTTGTGCGTGTCA |  |  |
| RmiR156e | GTCGTATCCAGTGCAGGGTCCGAGGTATTCGCACTGGATACGACGTGCTC |  | reverse transcription of miR156e |
| qmiR156e | CGCGCGTTGACAGAAGATAGA |  | qRT-PCR of miR156e |
| RmiR156g-5p | GTCGTATCCAGTGCAGGGTCCGAGGTATTCGCACTGGATACGACGTGCCC |  | reverse transcription of miR156g-5p |
| qmiR156g-5p | CGCGCGTTGACAGAAGATAGA |  | qRT-PCR of miR156g-5p |
| RmiR160c | GTCGTATCCAGTGCAGGGTCCGAGGTATTCGCACTGGATACGACTGGCAT |  | reverse transcription of miR160c |
| qmiR160c | CGTGCCTGGCTCCCTGA |  | qRT-PCR of miR160c |
| RmiR2587a | GTCGTATCCAGTGCAGGGTCCGAGGTATTCGCACTGGATACGACCAGGGT |  | reverse transcription of miR160c |
| qmiR2587a | CGCGTTGACCGTTCATATGA |  | qRT-PCR of miR5287a |
| Rnovel-110 | GTCGTATCCAGTGCAGGGTCCGAGGTATTCGCACTGGATACGACAGTCGA |  | reverse transcription of novel-miR110 |
| qnovel-110 | GCGAATCGAACTTGTCTGAGC |  | qRT-PCR of novel-miR110 |
| Rnovel-27 | GTCGTATCCAGTGCAGGGTCCGAGGTATTCGCACTGGATACGACGAGAAA |  | reverse transcription of novel-miR27 |
| qnovel_27 | CGCGCATGTGACCATATTAGAA |  | qRT-PCR of novel-miR27 |
| mQ-Primer-R | AGTGCAGGGTCCGAGGTATT |  | reverse primer of miRNA qRT-PCR |
| mRNA qRT-PCR | | | |
| actin-F | CACGAGACCACCTACAACTCTATC | JQ028730.1 |  |
| actin-R | CATACGGTCAGCAATACCTGG |  |  |
| qIOMT9-F | CGTTACCTCGCACACAATGG | MS.gene91606 |  |
| qIOMT9-R | AGCTCTGAAGCAACAGTGAGA |  |  |
| qSPL13-F | ATGAACACCGTCTCAACCCC | MS.gene74056 |  |
| qSPL13-R | GAATTTACCGTGTGCGGTGG |  |  |
| qARF18-F | TCAGGCGCGAATATGACTGG | MS.gene056152 |  |
| qARF18-R | ACTTGCGCGAGGGTAGTAAA |  |  |
| qPCKAF | GTGAATCGTTGGCGTCGTTG | MS.gene69100 |  |
| qPCKAR | ATCCACGTGTGCACTACTCC |  |  |
| qNRT3.1-F | CACGAACGCGGGTAAGAGTA | MS.gene48315 |  |
| qNRT3.1-R | ACCTAGGGACACAACCGAGA |  |  |
| qSPX-F | CACAAAGCACTTCGCCAACA | MS.gene066027 |  |
| qSPX-R | CAACCTGTAGACAACCCGACA |  |  |

**Supplementary Table 1** The primer sequences of miRNA reverse transcription, and miRNAs and target genes for qRT-PCR assay.
