# Supplementary figures and images for: Integrated mRNA and microRNA expression analysis of root response to phosphate deficiency in Medicago sativa
Source: Front Plant Sci. 2022 Sep 13;13:989048. doi: 10.3389/fpls.2022.989048 (PMC9513243; doi:10.3389/fpls.2022.989048)

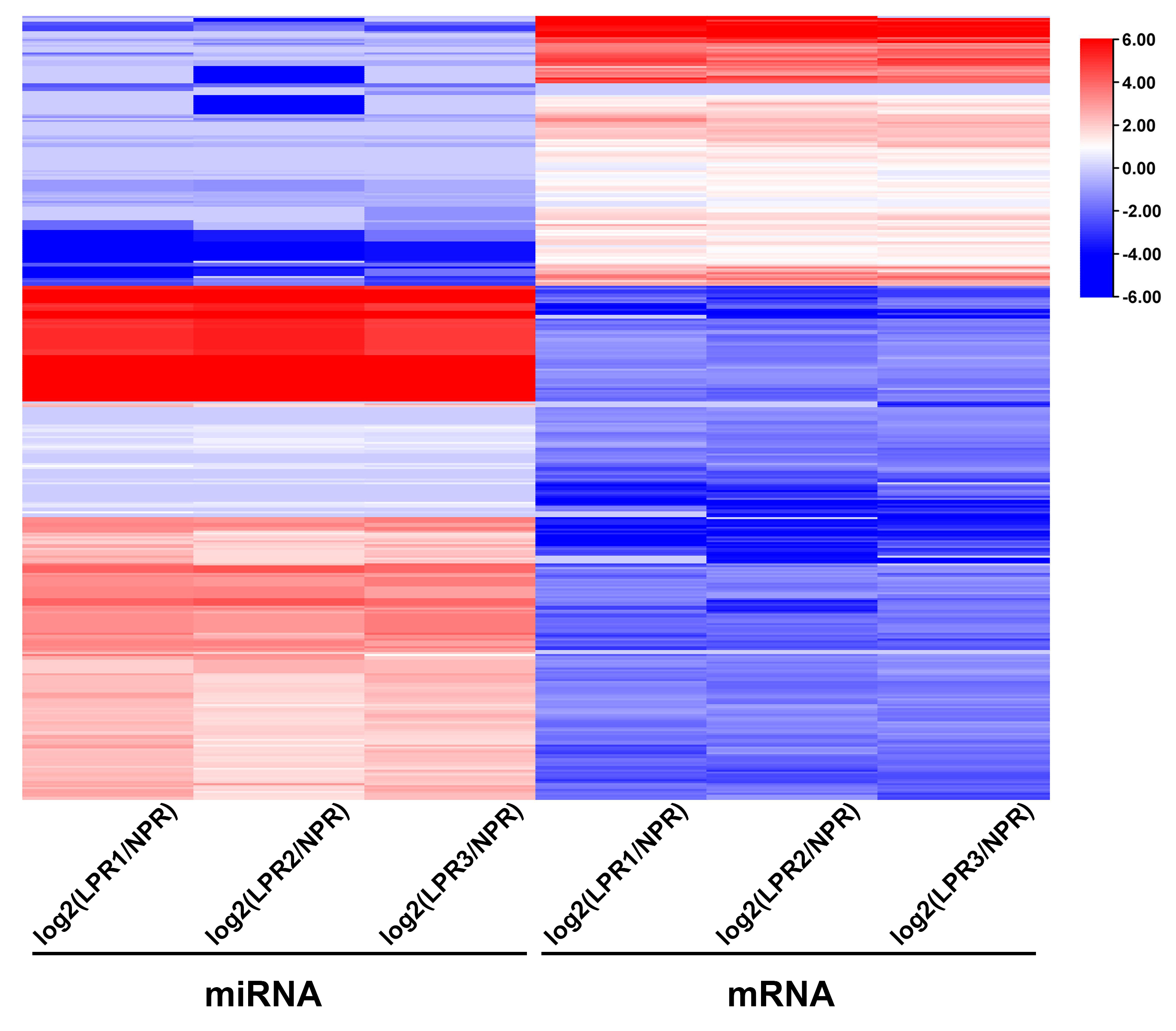

Supplement: SUPPLEMENTARY FIGURE 1 — MapMan enrichment of differentially expressed genes in alfalfa root under Pi deficiency. [file Image_1.JPEG]

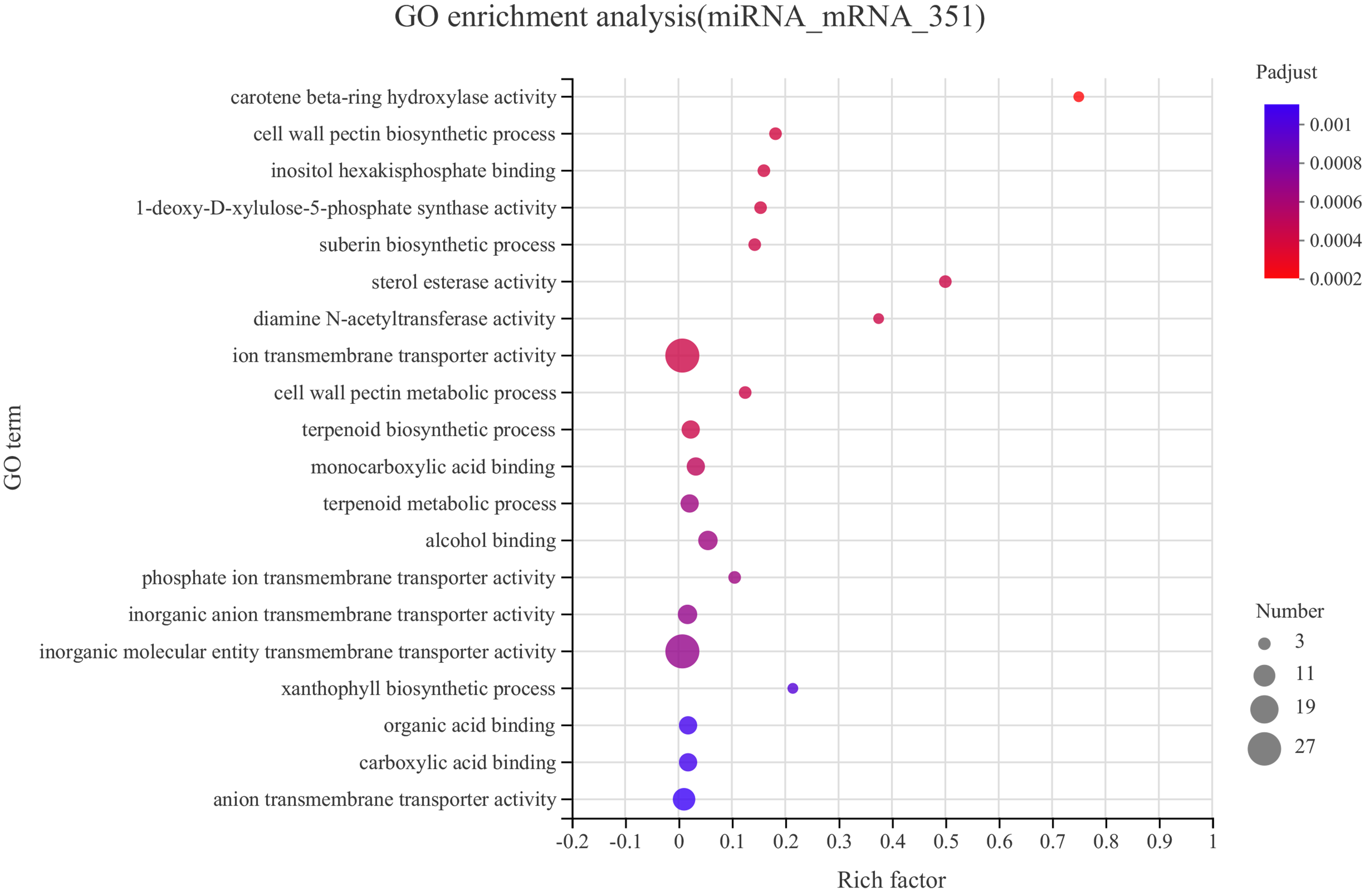

Supplement: SUPPLEMENTARY FIGURE 2 — The expression of miRNAs and their targets mRNA under Pi deficiency. [file Image_2.TIF]

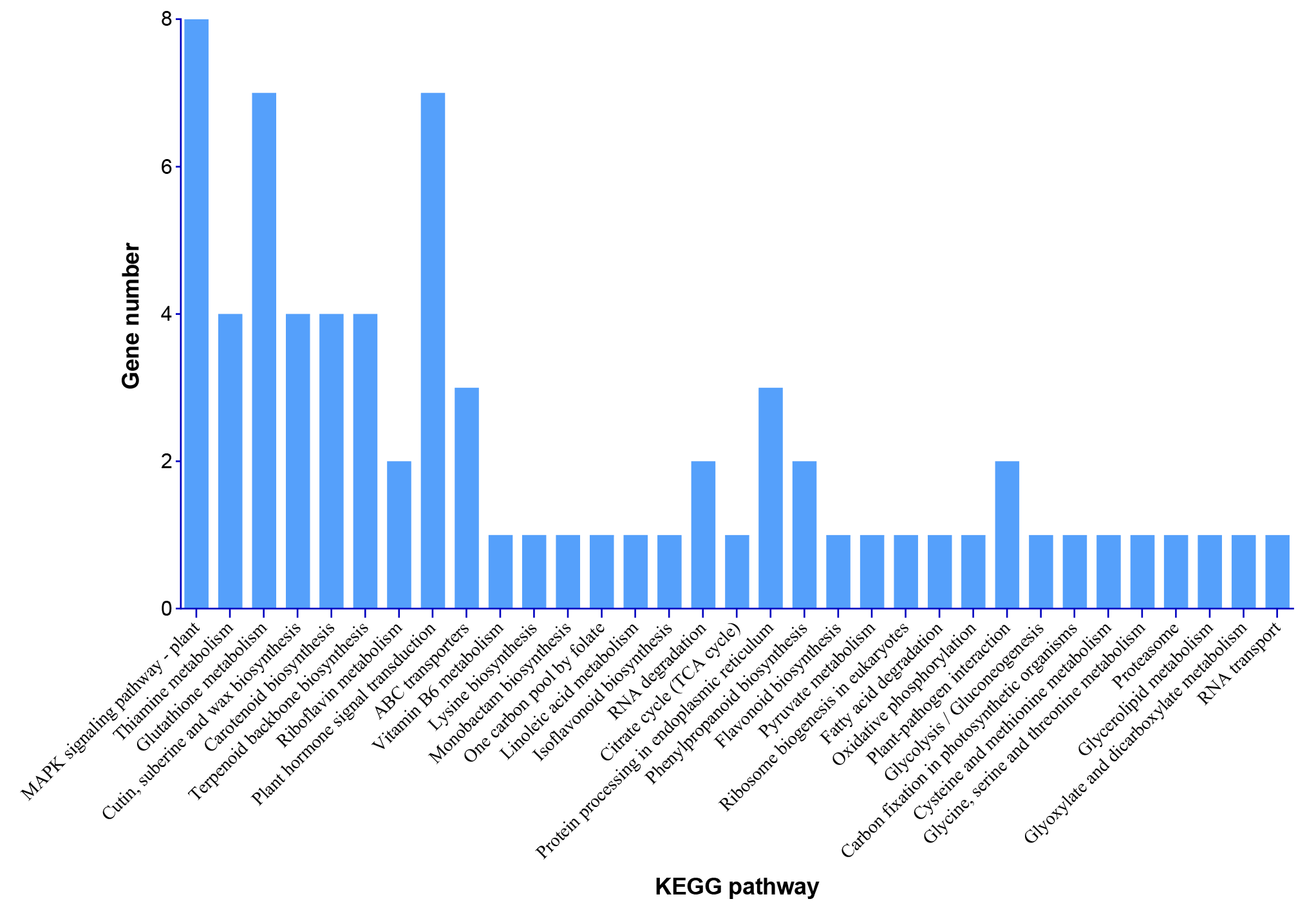

Supplement: SUPPLEMENTARY FIGURE 3 — Gene ontology enrichment of miRNA-targeted mRNAs under Pi deficiency. [file Image_3.TIF]

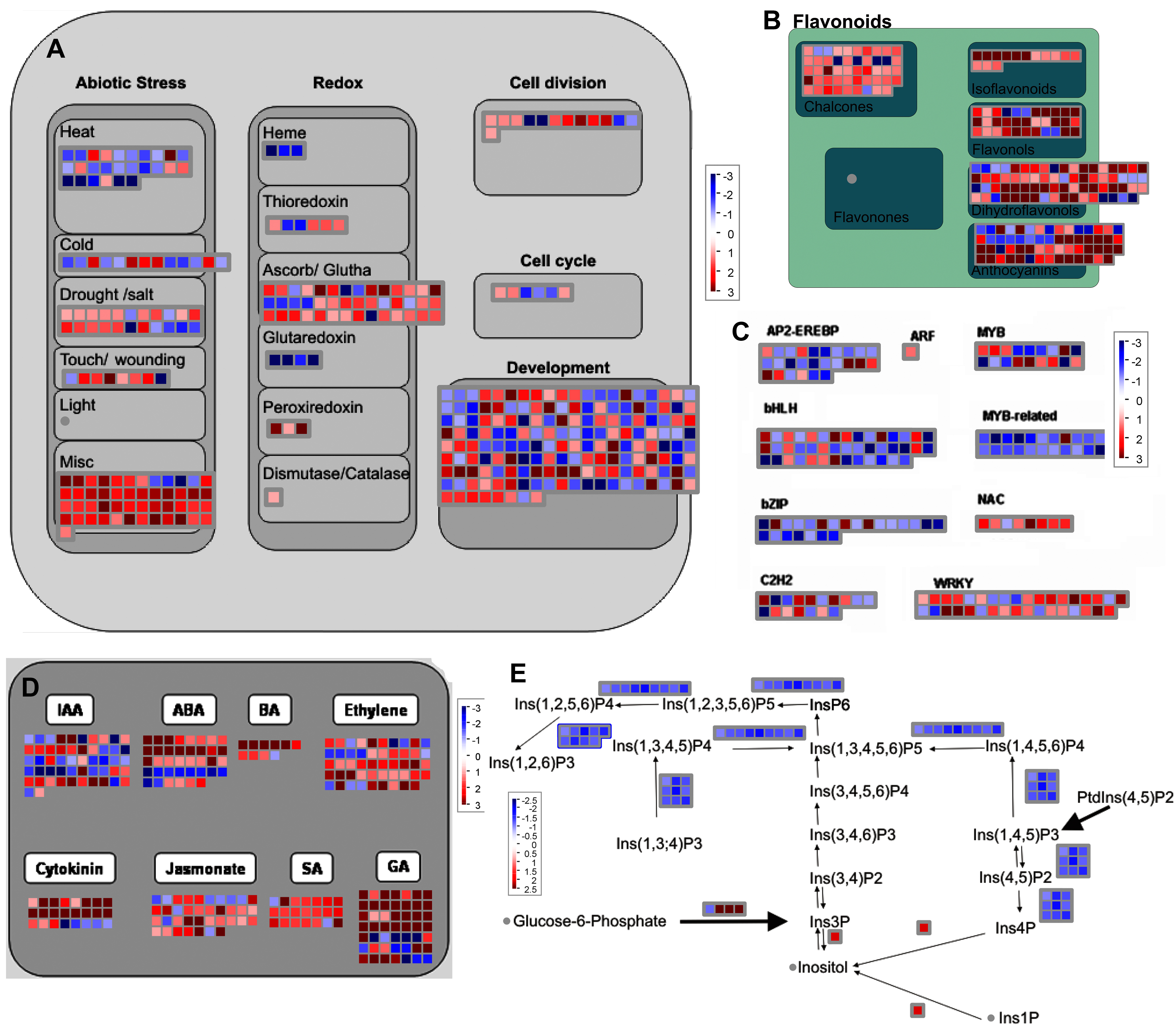

Supplement: SUPPLEMENTARY FIGURE 4 — Kyoto Encyclopedia of Genes and Genomes pathways of miRNA-targeted mRNAs under Pi deficiency. [file Image_4.JPEG]
